# Why Is Hamstring Strain Injury so Common in Sport Despite Numerous Prevention Methods? Are There Any Missing Pieces to This Puzzle?

**DOI:** 10.3389/fphys.2021.586624

**Published:** 2021-05-13

**Authors:** Łukasz Oleksy, Anna Mika, Jacek Pacana, Olimpia Markowska, Artur Stolarczyk, Renata Kielnar

**Affiliations:** ^1^Orthopaedic and Rehabilitation Department, Medical University of Warsaw, Warsaw, Poland; ^2^Oleksy Medical and Sports Sciences, Łańcut, Poland; ^3^Polish Strength and Conditioning Association, Wrocław, Poland; ^4^Institute of Clinical Rehabilitation, University of Physical Education in Kraków, Kraków, Poland; ^5^Faculty of Mechanical Engineering and Aeronautics, Rzeszów University of Technology, Rzeszów, Poland; ^6^Institute of Health Sciences, Medical College of Rzeszów University, Rzeszów, Poland

**Keywords:** hamstring, injury, sports, evaluation, prevention

Hamstring strain injury (HSI) is the most common non-contact injury representing 37% of all muscle traumas in professional sport (Alonso-Fernandez et al., 2018; Cuthbert et al., 2020). The recurrence rate of HSI ranging from 16 to 60% suggests that current protective training programs and rehabilitation strategies are not very effective (Brockett et al., 2001; Ribeiro-Alvares et al., 2018). Duhig et al. (2019) have found, that absolute training volumes were not associated with HSI, but exposure to transiently elevated load volumes, relative to those an athlete is regularly performing, increased the probability of hamstring injury.

During the terminal swing phase of the gait cycle, the hamstrings eccentrically decelerate knee extension and hip flexion before weight-bearing, when the biceps femoris (BF) long head reaches ~110% of its length (Schache et al., 2013). During these movements, eccentric hamstring contraction produces strains and microscopic damage to muscle fibers, which may provide a point of weakness resulting in a major tear (Brockett et al., 2001; Schache et al., 2013). However, it is not clear whether the hamstring contraction is fully eccentric or a quasi-isometric action (Cuthbert et al., 2020). Van Hooren and Bosch (2017) have suggested that elastic tissues could lengthen while the contractile element remains isometric. The most popular form of hamstring eccentric strengthening is Nordic Hamstring Exercise (NHE) (Cuthbert et al., 2020). In some studies, improvement in muscle strength and reduction of HSI have been demonstrated, suggesting its effectiveness for the functional eccentric training of athletes (Alonso-Fernandez et al., 2018; Ribeiro-Alvares et al., 2018). Despite its success in reducing HSI, the adoption of the NHE in elite sport still is poor (Bourne et al., 2017). It was suggested, that the NHE does not challenge the hamstrings at sufficient lengths to optimize injury prevention. The exercise resulted in greater fascicle lengthening are considered as more beneficial, so there are discrepancies between studies, if the NHE has purely eccentric or mixed nature (Bourne et al., 2017; Alonso-Fernandez et al., 2018; Ribeiro-Alvares et al., 2018). On the other hand, it has been reported that sprint training is more specific to the injury mechanism, and therefore, may be more adequate for increasing eccentric hamstring strength (Freeman et al., 2019). Some authors have stated that eccentric exercise produces some muscle fiber damage, which is accompanied by transient changes in the length-tension relation visible as a shift in a muscle's optimum torque angle curve that is suggested to be protection against further damage from eccentric exercise (Brockett et al., 2001). Additionally, isometric rather than eccentric hamstring exercises have been suggested as more specific, especially for runners (Van Hooren and Bosch, 2017).

It has also been reported that force production capabilities of the muscle and the velocity at which this occurs are influenced by muscle architecture such as pennation angle, muscle thickness and fascicle length (Alonso-Fernandez et al., 2018). However, the BF long head has shorter fascicles than the BF short head, potentially increasing the susceptibility of the BF long head to injury. It has been noted that the increase in eccentric hamstring strength may modify its architecture, potentially decreasing the risk of HSI (Alonso-Fernandez et al., 2018; Ribeiro-Alvares et al., 2018; Cuthbert et al., 2020).

Nonetheless, despite the positive effects of different prevention and rehabilitation programs, the percentage of HSI and its recurrence rate is still very high. This suggests, that they are not fully effective. A probable reason for this is that they are based on different methodological approaches while none of them take all the biomechanical aspects of this problem into account. There are many theories describing the causes of hamstring injuries, but some point is probably missing. It should be underlined that we live in a three-dimensional world, where all movements are performed on three planes.

The potential relationship between pelvis position and HSI was suggested by some authors. Higashihara et al. (2015), Mendiguchia et al. (2020), Mendiguchia et al. (2020) have reported that biceps femoris muscle is anatomically linked to the ischial tuberosity with connections to the sacrotuberous ligament and therefore closely related to changes in anterior pelvic tilt. Also, a greater pelvic anteversion and trunk flexion together with a greater elongation of the biceps femoris myotendinous junction have been shown, during both the late stance and late swing phase of sprinting (Higashihara et al., 2015). But, all recent studies were focused only on changes in anterior-posterior pelvic tilt, what may partially be related to increased HSI risk. There is a lack of studies which include the fact, that if the pelvis is rotated in relation to body axis, it may influence the whole gait pattern, also changing the function of the hamstring. As was previously reported, body axial rotation is common, and ~80 percent of people have rotated body patterns (Oleksy et al., 2019). Thus, the pattern of rotation and fascial tension in specific body parts may cause the body to be more prone to asymmetry. Therefore, we have suggested that the effect of lumbo-pelvic complex rotation on hamstring muscle function may be a missing element of the low effectiveness of HSI treatment, and a key point in its high recurrence.

Another important issue is that hamstring muscles work differently depending on gait cycle phase. It has been reported that the BF operates as a hip extensor and adductor as well as a knee flexor, abductor and external-rotator (Schache et al., 2013). All these functions can be disrupted when free pelvis movement during gait is blocked due to its axial rotation.

In our previous study (Oleksy et al., 2019), it was reported that lumbo-pelvic complex misalignment has significant impact on pelvic floor muscle function. Also, the model of strain forces acting on the BF muscle presented in this work, suggests some impact of pelvis rotation on the behavior of these muscles (Figure 1). It has been shown that pelvis torsion may increase the strain, especially on the BF long head with the highest values at the proximal and distal attachment points (Figure 1, Supplementary Material). What is more, the strain forces are asymmetrically present (Figure 1, Supplementary Material). Therefore, exercises like NHE or sprint training, when applied symmetrically, may expose the BF, on one side, to increased amount of eccentric mictro-tears. We have suggested that excessive rotation of the lumbo-pelvic complex in relation to body axis may disturb the biomechanics of the whole gait pattern. Therefore, it should be considered as the missing puzzle piece in the prevention and treatment of HSI. A practical implication of this observation is that coaches and clinicians, in their daily routine, should perform the evaluation of lumbo-pelvic complex alignment, even using simple functional tests and incorporating appropriate exercise so that the BF long head may obtain optimal length on the stretched side, and thus, the right conditions for effective eccentric reinforcement.

**Figure 1:**
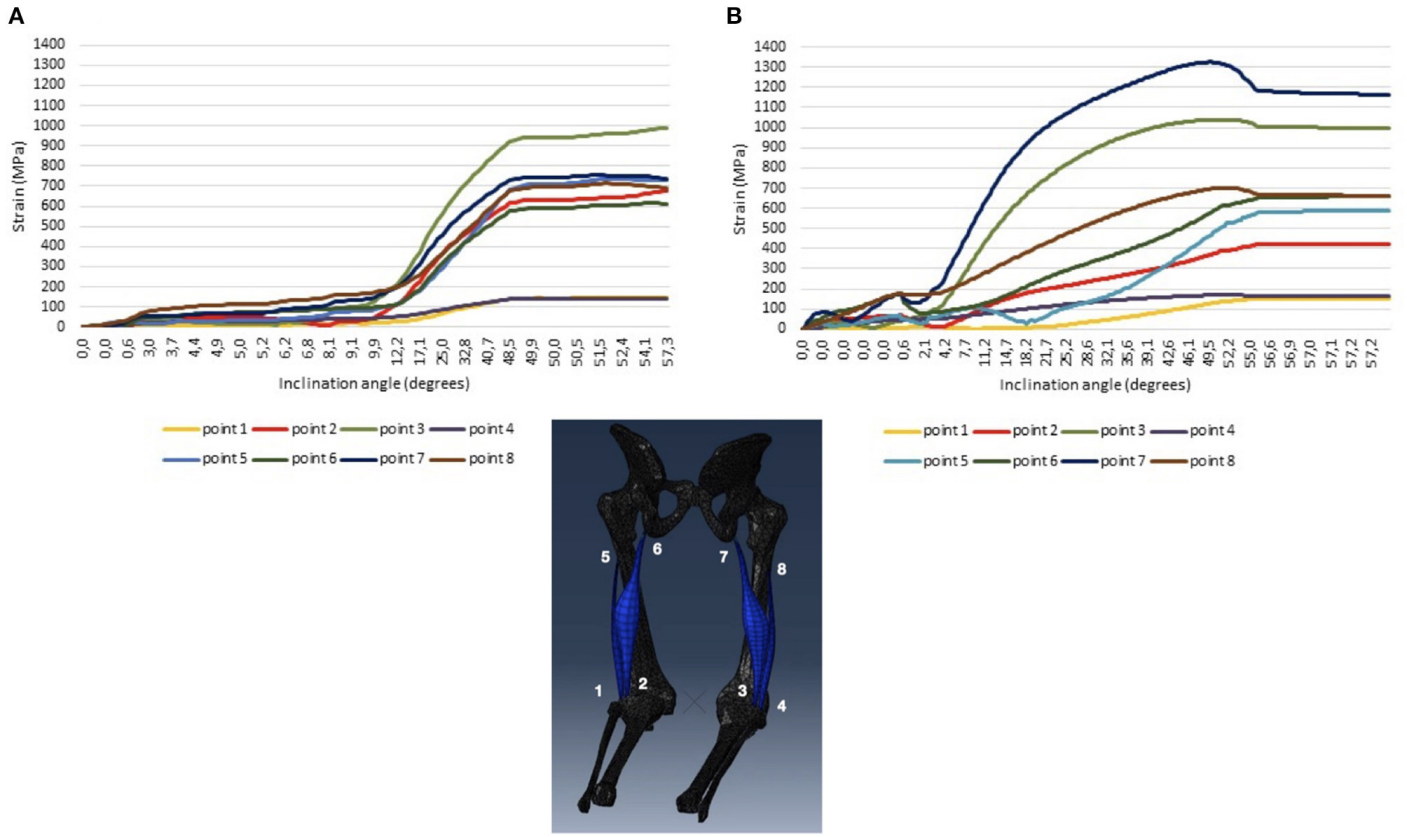
Strain forces acting on the biceps femoris muscle long and short heads during movement with lumbo-pelvic complex rotation in relation to body axis 0.1 radian **(A)** and 0.4 radian **(B)**.

## Author Contributions

ŁO and AM: study concept and design, data analyses and interpretation, literature search, writing and editing the manuscript. JP and OM: data collection. AS and RK: study concept and design, data interpretation, editing the manuscript. All authors contributed to the article and approved the submitted version.

## Conflict of Interest

The authors declare that the research was conducted in the absence of any commercial or financial relationships that could be construed as a potential conflict of interest.

## Supplementary Material

Supplementary Video 1 Strain forces acting on the biceps femoris muscle long and short heads during movement with lumbo-pelvic complex rotation in relation to body axis 0.1 radian.

Supplementary Video 2 Strain forces acting on the biceps femoris muscle long and short heads during movement with lumbo-pelvic complex rotation in relation to body axis 0.4 radian.
